# Preoperatively elevated RDW-SD and RDW-CV predict favorable survival in intrahepatic cholangiocarcinoma patients after curative resection

**DOI:** 10.1186/s12893-021-01094-6

**Published:** 2021-03-01

**Authors:** Xingchen Li, Qichen Chen, Xinyu Bi, Jianjun Zhao, Zhiyu Li, Jianguo Zhou, Zhen Huang, Yefan Zhang, Rui Mao, Hong Zhao, Jianqiang Cai

**Affiliations:** Department of Hepatobiliary Surgery, National Cancer Center/National Clinical Research Center for Cancer/Cancer Hospital, Chinese Academy of Medical Sciences and Peking Union Medical College, No. 17 Nanli, Panjiayuan, Chaoyang District, Beijing, China

**Keywords:** Intrahepatic cholangiocarcinoma, Liver resection, RDW, Prognosis

## Abstract

**Background:**

Recent studies suggest red blood cell distribution width (RDW) was a prognostic factor in various types of cancer patients, although the results are controversial. The objective of this study was to investigate the significance of RDW in patients with intrahepatic cholangiocarcinoma (ICC) after radical resection.

**Method:**

The relationship between the preoperative serum RDW value and clinic pathological characteristics was analyzed in 157 ICC patients between January 2012 and June 2018 who underwent curative resection. X-tile software was used to determine 40.2 fl, 12.6% as the optimal cut-off value for RDW-SD and RDW-CV respectively. 153 patients were classified into the low RDW-SD (≤ 40.2, n = 53) group and the high RDW-SD (> 40.2, n = 104) group, low RDW-CV (≤ 12.6, n = 94) group and the high RDW-CV (> 12.6, n = 63). Based on the RDW-SD combined with RDW-CV (SCC), classified into SCC = 0, 1 and 2 group. Kaplan–Meier survival analysis and Cox proportional hazard models were used to examine the effect of RDW on survival.

**Results:**

Kaplan–Meier curve analysis showed that Patients with RDW-SD > 40.2 were significantly associated with better OS (P = 0.004, median OS: 68.0 months versus 17.0 months). Patients with RDW-CV > 12.6 were significantly associated with better OS (p = 0.030, median OS: not reach versus 22.0 months). Compared with a SCC = 0 or SCC = 1, SCC = 2 was significantly associated with better OS (p < 0.001, median OS: not reach versus 33.0 months versus 16, respectively). In the multivariate analysis, RDW-SD > 40.2 fl (HR = 0.446, 95% CI: 0.262–0.760, p = 0.003), RDW-CV > 12.6% (HR = 0.425, 95%CI: 0.230–0.783, p = 0.006), SCC = 2 (HR = 0.270, 95%CI: 0.133–0.549, p < 0.001) were associated with favorable OS. The multivariate analysis showed RDW-SD, RDW-CV and SCC level were not independent prognostic factors for DFS.

**Conclusions:**

Preoperative low levels of RDW are associated with poor survival in ICC after curative resection. This provides a new way for predicting the prognosis of ICC patients and more targeted intervention measures.

## Background

Intrahepatic cholangiocarcinoma (ICC) is a relatively rare type of primary liver cancer that arises from the intrahepatic bile duct epithelium and accounts for 75% of primary liver carcinomas, the incidence of which is increasing year by year [1]. The only curative treatment is surgical resection. Even when potentially curative resections are achieved, the 5-year survival rate after resection is only 8 to 47% because these tumors have a high degree of malignancy, insidious onset and early subclinical changes and are very difficult to discover. Clear margins during resection are emphasized as the most important factor for good local control and a favorable prognosis; in addition, lymph node metastasis is also one of the most significant prognostic factors for survival in ICC [2]. Surgery requires radical resection with appropriate lymph node resection. The prognosis of patients is closely related to whether radical resection can be performed, but the radical resection rate of ICC is only 15 to 20%, far lower than the surgical resection rate of 70% for distal cholangiocarcinoma. Even though patients with ICC who undergo extended resection still have a poor prognosis, this is closely related to the high incidence of local recurrence and distant metastasis [3]. To date, no systemic adjuvant therapy has improved overall survival (OS), despite the increased research effort and active clinical trials investigating a variety of drugs [4]. We should attach great importance to this kind of malignant disease in the clinic.

Although the reasons for the high recurrence rate in ICC are complicated, inflammation plays an important role in the malignant progression and metastasis of ICC [5, 6]. In addition, there is substantial evidence that systemic inflammation predicts survival and recurrence of ICC after resection [7]. Some studies have shown that systemic inflammatory markers of serum parameters, including platelet count (PLT), hemoglobin, neutrophil–lymphocyte ratio (NLR) and platelet-lymphocyte ratio (PLR), can predict the survival of a variety of human cancers [8–12]. Red cell volume distribution width (RDW) is a conventional biomarker of erythrocyte volume variability and an indicator of homeostasis [13]. Recent evidence suggests that unequal erythrocyte action is involved in a variety of human diseases, such as cardiovascular disease [14, 15] and cancer [16, 17]. There is some evidence that high RDW levels are a negative prognostic indicator for these diseases, and inflammation is the mechanism leading to these high levels [13]. There is increasing evidence that elevated RDW levels are also associated with poor prognosis in a variety of cancers, including hepatocellular carcinoma (HCC) [18, 19], esophageal cancer [20, 21], lung cancer [22] and hematological malignancies [23]. However, a review of the previous related literature shows some research limitations. Multivariate analysis showed that preoperative RDW is not an independent prognostic indicator of OS in gastric adenocarcinoma patients [24]. Due to the inevitable heterogeneity of the study samples, the prognostic effects of RDW have not been fully investigated. The predictive value of RDW in ICC patients has not been demonstrated. The purpose of this study was to evaluate the relationship between RDW and clinical outcomes in ICC patients.

## Methods

### Patients

The clinical date of 157 cases with ICC from our hospital between January 2012 and June 2018 were collected and analyzed retrospectively. The inclusion criteria were as follows: (1) complete (R0) resection of liver cancer and histopathological diagnosis of ICC; and (2) none of these patients had previous malignant disease. The exclusion criteria were as follows: (1) patients with clinical or pathologic distant metastasis; (2) perioperative mortality; (3) no follow-up data; and (4) pretreatment diseases associated with RDW levels (thrombosis, sepsis, cardiovascular disease, etc.). The Ethics Committee of the Cancer Hospital, Chinese Academy of Medical Sciences approved the study, and the requirement for informed consent was waived.

Major resections were defined as a resection of more than two segments, and other resection was described as a minor resection. Serum red blood cell distribution width-standard deviation (RDW-SD) levels and red blood cell distribution width- coefficient of variation (RDW-CV) levels were measured within 1 week before surgery. Blood samples for the evaluation of serum RDW-SD levels (37.0–57.0 fl) and RDW-SD levels (111.6–14.6 fl) were obtained by using peripheral venous punctures. The RDW-SD combined with RDW-CV (SCC) was established to analyses the prognostic value of survival. The SCC was scored as 0 (decreased RDW-SD levels with decreased RDW-CV), 2 (elevated RDW-SD levels with elevated RDW-SD levels), or 1 (all other combinations). Each postoperative complication was described as a severity grade using the Clavien–Dindo classification system (I–V). Major complications were classified as Clavien–Dindo III–V. If multiple postoperative complications occurred in one patient, the higher grade was used.

### Follow-up

Patients were required to visit the clinics 1 month after surgery. Then, the patients were required to visit the clinics every 3 months for the next 2 years, every 6 months for the following 3 years, and once annually thereafter. Patients received postoperative serum tumor markers (carbohydrate antigen 19-9 [CA19-9], carcinoembryonic antigen [CEA] and alpha fetoprotein [AFP]) measurements and magnetic resonance imaging (MRI) or computed tomography (CT) at every visit time. Recurrence was detected by the tumor markers level and imaging. The patients’ follow-up adjuvant treatment was chosen based on the first review, which included assessments of pathological stage, microvascular invasion (MVI) and other high-risk relapse factors. During the follow-up review process, when recurrence was confirmed, salvage treatment including reoperation, percutaneous ablation or transarterial chemoembolization (TACE) was performed as needed. In this study, the examination data of the patients were extracted from the hospital medical records system, and the patients were followed up by telephone. The deadline for follow-up was the date of the last follow-up or death.

### Statistical analysis

The clinicopathologic characteristics were compared using the X^2^ and Mann–Whitney U tests, as appropriate. For testing markers (albumin [ALB], total bilirubin [TBIL], aspartate aminotransferase [AST], alanine aminotransferase [ALT], et al.), the upper limit of normal value was defined as cutoff value; for operation time and intraoperative blood loss, the median was defined as cutoff value. X-tile [25] analysis was implemented to investigate the optimal cut-off point of RDW-SD and RDW-CV. The X-tile plots are created by dividing marker data into two populations: low and large. All possible divisions of the marker data are assessed. Associations can be calculated at each division by a variety of standard statistical tests, including the log-rank test for survival and means tests for associations between other marker data. Alternatively, the program can select the optimal division of the data by selecting the highest X^2^ value. OS was defined as the interval between the date of resection and the date of death or the last follow-up, and disease-free survival (DFS) was defined as the duration from resection to tumor progression or the last follow-up. This study used the Kaplan–Meier method to estimate DFS and OS and statistically compared the data by log-rank test. A forward LR Cox regression model was created to identify prognostic factors that influence OS and DFS. Variables with P < 0.10 in the univariable analysis were included in the multivariable analysis. The RDW-SD level and RDW-CV level exhibited collinearity (p < 0.001). To prevent collinearity, RDW-SD was included in the multivariate analysis of Model 1 (exclude of RDW-CV), and RDW-CV was included in the multivariate analysis of Model 2 (exclude of RDW-SD). Because the SCC score was based on RDW-SD and RDW-CV, the multivariate analysis of Model 3 included SCC score and factors with a p < 0.1 in the univariate analysis exclude of the RDW-SD and RDW-CV. All analyses were performed using SPSS, version 22 software (Armonk, NY, USA). p < 0.05 was considered statistically significant.

## Results

### Clinicopathological characteristics

The median age of all 157 patients was 58.00 (IQR: 51.50–64.00) years and most patients (55.4%) were male. The proportion of patients with positive serum hepatitis B surface antigen (HBsAg) was 21.7%. The proportion of patients with lesions in the central liver was 49.7%. The median diameter of the largest ICC lesion was 5.5 (IQR 3.8–7.0) cm, and 54.8% of patients had a lesion larger than 5 cm. A total of 19.1% of patients had multiple tumors and 26.8% of the patients had LNM. Ninety one patients (58.0%) were observed with poorly differentiated tumors.

The preoperative clinical laboratory tests were as follows: tumor markers: preoperative CA19-9 > 27 U/mL (52.4%), preoperative CEA > 5 ng/mL (22.3%); liver function markers: ALB ≥ 40 g/L (79.0%), TBIL > 21 µmol/L (11.5%), AST > 40 U/L (9.6%), and ALT > 50 U/L (8.9%). The median RDW-SD level was 41.1 fl (IQR 39.7–43.3). The median RDW-CV level was 12.4% (IQR 12.0–13.1). A total of 58.6% (92/157) of the patients have postoperative complications, including 33 major complications and 59 minor complications. Seventy-nine patients had an operation time ≥ 230 min, and 49.0% patients had blood loss ≥ 300 mL. Ten patients (6.4%) received preoperative therapy and 66 patients (42.0%) received postoperative therapy. The detailed clinicopathologic parameters of patients are in Table 1.

**Table 1:** Patient and tumour characteristics

Item	All patients (n = 157)(%)	RDW-SD > 40.2 fl VS RDW-SD ≤ 40.2 fl	RDW-CV > 12.6% VS RDW-CV ≤ 12.6%	SCC = 0 VS SCC = 1 VS SCC = 2
		p	p	p
Age ≥ 60 years	70 (44.6)	0.056	0.494	0.029
Male	87 (55.4)	0.900	0.977	0.882
BMI ≥ 24 kg/m^2^	94 (60.3)	0.355	0.187	0.392
ASA score 3–4	13 (8.3)	0.027	0.190	0.099
Cirrhosis	57 (36.3)	0.790	0.020	0.279
HBsAg ( +)	34 (21.7)	0.294	0.758	0.906
T3-T4 stage	33 (21.0)	0.768	0.005	0.008
Lymphnode metastasis	42 (26.8)	0.971	0.736	0.781
Tumor size ≥ 5 cm	86 (54.8)	0.314	0.873	0.854
Multiple tumors	30 (19.1)	0.357	0.213	0.067
Tumor location (Central tumor)	78 (49.7)	0.573	0.580	0.994
Tumor location (right lobe)	77 (49.0)	0.970	0.785	0.721
Poorly differentiation	91 (58.0)	0.089	0.515	0.275
Preoperative CA19-9 > 27 U/mL	82 (52.2)	0.333	0.279	0.374
Preoperative CEA > 5 ng/mL	35 (22.3)	0.174	0.142	0.202
RDW-SD > 40.2 fl	104 (66.2)	–	< 0.001	–
RDW-CV > 12.6%	63 (40.1)	< 0.001	–	–
SCC = 0	46 (29.3)	–	–	–
SCC = 1	55 (35.0)	–	–	–
SCC = 2	56 (35.7)	–	–	–
ALB ≥ 40 g/L	124 (79.0)	0.375	0.133	0.283
TBIL > 21 umol/L	18 (11.5)	0.968	0.015	0.169
AST > 40 U/L	15 (9.6)	0.591	0.587	0.179
ALT > 50U/L	14 (8.9)	0.053	0.724	0.024
Operation time ≥ 230 min	79 (50.3)	0.027	0.896	0.052
Blood loss ≥ 300 ml	77 (49.0)	0.921	0.297	0.827
Major liver resection	113 (72.0)	0.667	0.548	0.812
Post-operative complications	92 (58.6)	0.164	0.720	0.353
Preoperative therapy	10 (6.4)	0.101	0.008	0.010
Adjuvant therapy	66 (42.0)	0.924	0.407	0.105

### Relationships among RDW-SD, RDW-CV, SCC and clinicopathological characteristics

X-tile software was used to determine 40.2 fl and 12.6% as the optimal cut-off values for RDW-SD and RDW-CV, respectively. Based on RDW-SD levels, 157 patients were classified into a low RDW-SD (≤ 40.2, n = 53) group and high RDW-SD (> 40.2, n = 104) group. Based on RDW-CV levels, 157 patients were classified into a low RDW-CV (≤ 12.6, n = 94) group and high RDW-CV (> 12.6, n = 63) group. Based on the RDW-SD combined with RDW-CV (SCC), 46 patients were classified into the SCC = 0 group, 55 patients were classified into the SCC = 1 group, and 56 patients were classified into the SCC = 2 group.

The high RDW-SD group had more patients with an ASA status of 1–2 (p = 0.027) and operation time < 230 min (p = 0.027) than the low RDW-SD group. The high RDW-CV group had more patients with non-liver cirrhosis (p = 0.020), T1-T2 stage disease (p = 0.005) and TBIL ≤ 21 µmol/L (p = 0.015). The patients with SCC = 0 was associated with an age < 60 years (p = 0.029) (Table 1).

### Prognostic value of RDW-SD, RDW-CV and SCC for survival

The median follow-up time was 33.00 months. The median OS and median DFS were 28.00 months (95% CI: 12.9–43.1) and 10.00 months (95% CI: 7.2–12.8), respectively. One hundred and nine patients (69.4%) underwent recurrence, and 73 patients (46.5%) died. The 1-, 3- and 5-year progression-free survival rates were 41.8%, 29.5%, and 20.9%, respectively. The 1-, 3- and 5-year survival rates were 72.9%, 46.0% and 41.1%, respectively.

**Fig. 1:**
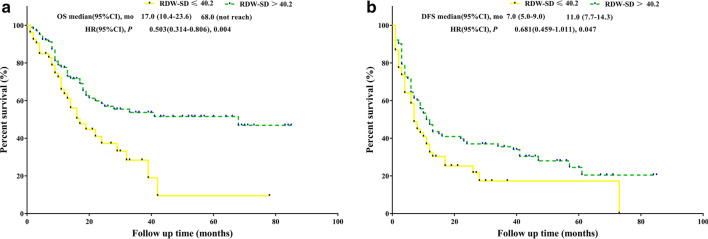
**a** OS analysis of RDW-SD > 40.2 fl versus RDW-SD ≤ 40.2 fl. **b** DFS analysis of RDW-SD > 40.2 fl versus RDW-SD ≤ 40.2 fl

**Fig. 2:**
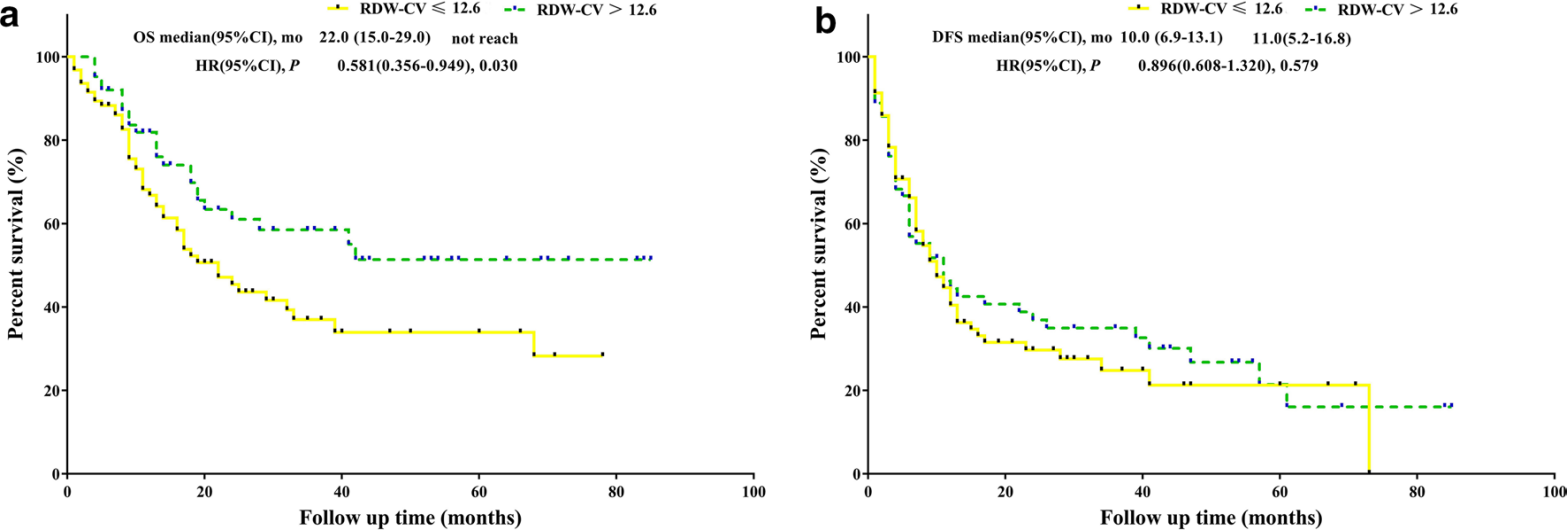
**a** OS analysis of RDW-CV > 12.6% versus RDW-CV ≤ 12.6% **b** DFS analysis of RDW-CV > 12.6% versus RDW-CV ≤ 12.6%

**Fig. 3:**
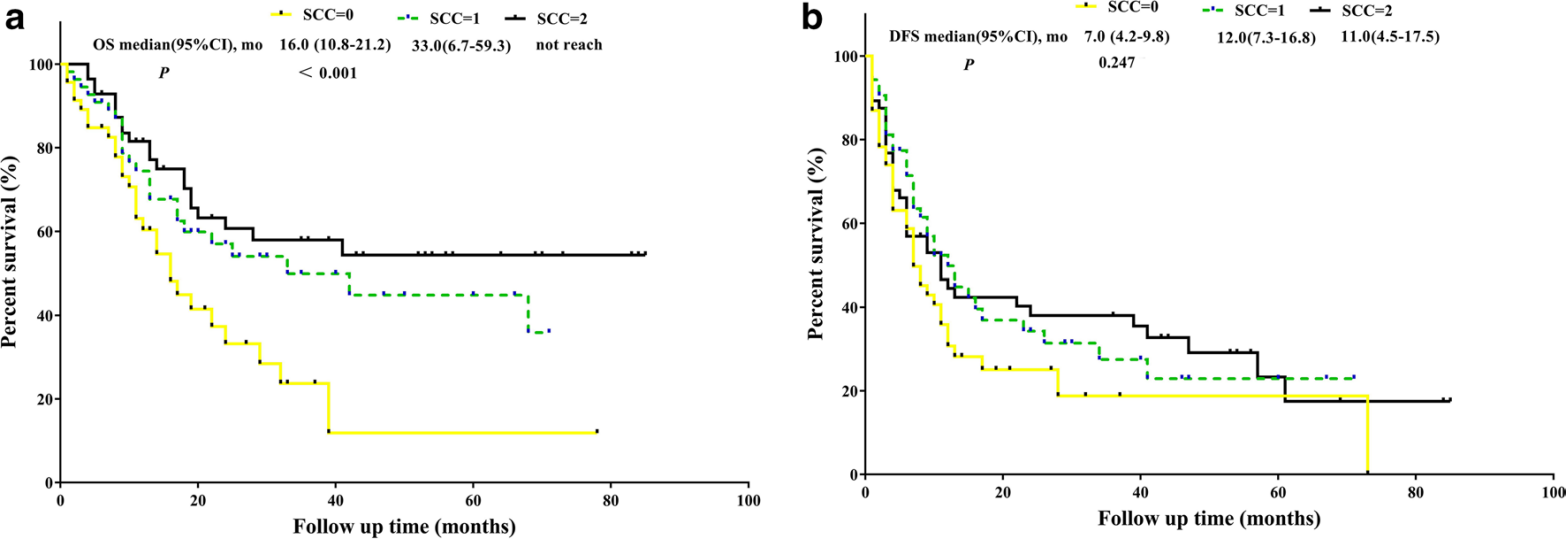
**a** OS analysis of SCC = 0 versus SCC = 1 versus SCC = 2 **b** DFS analysis of SCC = 0 versus SCC = 1 versus SCC = 2

Kaplan–Meier curve analysis revealed that patients with RDW-SD > 40.2 were significantly associated with better OS (p = 0.004, median OS: 68.0 months versus 17.0 months) and better DFS (p = 0.047, median DFS: 11.0 months versus 7.0) than those with low RDW-SD values (Fig. 1). Patients with RDW-CV > 12.6 were significantly associated with better OS (p = 0.030, median OS: not reached versus 22.0 months) and had an equivalent DFS (p = 0.579, median DFS: 11.0 months versus 10.0) than those with low RDW-CV values (Fig. 2). Compared with patients with SCC = 0 or SCC = 1, patients with SCC = 2 were significantly associated with better OS (p < 0.001, median OS: did not reach versus 33.0 months versus 16, respectively), but all three SCC values had an equivalent DFS (p = 0.247, median DFS: 11.0 months versus 12.0 months versus 7.0 months, respectively) (Fig. 3).

### OS analysis

In the univariate analysis, T3-T4 stage (p < 0.001), lymph node metastasis (p < 0.001), noncentral tumor (p = 0.027), poor differentiation (p = 0.044), preoperative CEA > 5 ng/mL (p = 0.001), RDW-SD ≤ 40.2 fl (p = 0.004), RDW-CV ≤ 12.6% (p = 0.030), ALB < 40 g/L (p = 0.048), operation time ≥ 230 min (p = 0.015), blood loss ≥ 300 ml (p = 0.030) and decreased SCC score (p = 0.007) were all associated with shorter OS (Table 2). In the multivariate analysis of model 1, RDW-SD > 40.2 fl (HR = 0.446, 95% CI: 0.262–0.760, p = 0.003), central tumor (HR = 0.367, 95% CI: 0.210–0.641, p < 0.001), and adjuvant therapy (HR = 0.423, 95% CI: 0.232–0.774, p = 0.005) were significantly associated with favorable OS. In the multivariate analysis of model 2, RDW-CV > 12.6% (HR = 0.425, 95% CI: 0.230–0.783, p = 0.006), central tumor (HR = 0.307, 95% CI: 0.171–0.552, p < 0.001), and adjuvant therapy (HR = 0.481, 95% CI: 0.264–0.876, p = 0.017) were significantly associated with favorable OS. In the multivariate analysis of model 3, the multivariate analysis showed that compared with SCC = 0, SCC = 1 (HR = 0.296, 95% CI: 0.153–0.571, p < 0.001) and SCC = 2 (HR = 0.270, 95% CI: 0.133–0.549, p < 0.001) were associated with favorable OS (Table 3).

**Table 2:** Prognostic factors for OS for ICC patients in univariate analysis

Factor	Univariate analysis		Factor	Univariate analysis	
	HR (95%CI)	Value p		HR (95%CI)	Value p
Age ≥ 60 years	0.778 (0.483–1.251)	0.300	Preoperative CEA > 5 ng/mL	2.365 (1.429–3.913)	0.001
Male	1.418 (0.887–2.267)	0.144	RDW-SD > 40.2 fl	0.503 (0.314–0.806)	0.004
BMI ≥ 24 kg/m^2^	1.356 (0.843–2.182)	0.209	RDW-CV > 12.6%	0.581(0.356–0.949)	0.030
ASA score 3–4	0.970 (0.420–2.238)	0.943	ALB ≥ 40 g/L	0.600 (0.361–0.996)	0.048
Cirrhosis	0.929 (0.575–1.500)	0.763	TBIL > 21 umol/L	0.913 (0.438–1.904)	0.808
HBsAg ( +)	0.754 (0.420–1.351)	0.343	AST > 40 U/L	1.773 (0.909–3.458)	0.093
T3-T4 stage	2.381 (.431–3.962)	0.001	ALT > 50U/L	1.814 (0.899–3.660)	0.096
Lymphnode metastasis		< 0.001	Operation time ≥ 230 min^b^	1.785(1.118–2.851)	0.015
Undissected lymph node	Reference	–	Blood loss ≥ 300 ml^b^	1.713 (1.053–2.788)	0.030
Negative lymph node	0.742 (0.414–1.331)	0.317	Major liver resection	1.419 (0.832–2.419)	0.198
Positive lymph node	2.808(1.554–5.072)	0.001	Post-operative complications	1.027 (0.640–1.647)	0.913
Tumor size ≥ 5 cm	1.529 (0.956–2.448)	0.077	Preoperative therapy	0.648 (0.204–2.060)	0.462
Multiple tumors	1.657(0.955–2.874)	0.073	Adjuvant therapy	0.663 (0.410–1.072)	0.094
Tumor location (central tumor)	0.587 (0.366–0.941)	0.027	*The prognostic value on SCC score*		0.007
Tumor location (right lobe)	0.858 (0.588–1.252)	0.427	SCC = 0	Reference	–
Poor differentiation	1.667 (1.013–2.743)	0.044	SCC = 1	0.554 (0.320–0.961)	0.035
Preoperative CA19-9 > 27 U/mL	1.332 (0.763–2.327)	0.313	SCC = 2	0.411 (0.231–0.730)	0.002

**Table 3:** Prognostic factors for OS for ICC patients in multivariate analysis

Factor	Multivariate analysis^a^				Factor	Multivariate analysis^a^			
	Model 1		Model 2			Model 1		Model 2	
	HR (95%CI)	Value p	HR (95%CI)	Value p		HR (95%CI)	Value *P*	HR (95%CI)	Value p
Age ≥ 60 years	–	–	–	–	RDW-SD > 40.2 fl	0.446 (0.262–0.760)	0.003	–	–
Male	–	–		–	RDW-CV > 12.6%	–	–	0.425 (0.230–0.783)	0.006
BMI ≥ 24 kg/m^2^	–	–	–	–	ALB ≥ 40 g/L	0.472 (0.257–0.866)	0.015	0.511 (0.269–0.974)	0.041
ASA score 3–4	–	–	–	–	TBIL > 21 umol/L	–	–	–	–
Cirrhosis	–	–	–	–	AST > 40 U/L	–	–	–	–
HBsAg ( +)	–	–	–	–	ALT > 50U/L	–	–	2.332 (0.984–5.528)	0.055
T3–T4 stage	–	–	–	–	Operation time ≥ 230 min^b^	1.894 (1.097–3.270)	0.019	1.804 (1.030–3.161)	0.039
Lymphnode metastasis	–	< 0.001	–	< 0.001	Blood loss ≥ 300 ml^b^	–	–	–	–
Undissected lymph node	Reference		Reference		Major liver resection	–	–	–	–
Negative lymph node	1.160 (0.581–2.318)	0.674	1.226 (0.595–2.526)	0.580	Post-operative Complications	–	–	–	–
Positive lymph node	3.715 (1.721–8.019)	0.001	4.394 (1.950–9.900)	< 0.001	Preoperative therapy	–	–	–	–
Tumor size ≥ 5 cm	–	–	2.041 (1.134–3.673)	0.017	Adjuvant therapy	0.423 (0.232–0.774)	0.005	0.481 (0.264–0.876)	0.017
Multiple tumors	–	–	–	–		*Multivariate analysis*^*c*^* (Model 3)*			
Tumor location (central tumor)	0.367 (0.210–0.641)	< 0.001	0.307 (0.171–0.552)	< 0.001		*HR (95%CI)*	*Value p*		
Tumor location (right lobe)	–	–	–	–	*The prognostic value on SCC score*^*c*^		< 0.001		
Poor differentiation	–	–	–	–	SCC = 0	Reference			
Preoperative CA19-9 > 27 U/mL	–	–	–	–	SCC = 1	0.296 (0.153–0.571)	< 0.001		
Preoperative CEA > 5 ng/mL	2.486 (1.305–4.734)	0.006	2.234 (1.164–4.287)	0.016	SCC = 2	0.270 (0.133–0.549)	< 0.001		

^a^To prevent colinearity, RDW-SD was included in the multivariate analysis of Model 1, and RDW-CV was included in the multivariate analysis of Model 2, respectively

^b^The median operation time and the median blood loss were chosen as the cut-off point

^c^Because the SCC score was based on the RDW-SD and RDW-CV, the multivariate analysis of prognostic value of SCC score included factors with a *P* < 0.1 in univariate analysis exclude of the RDW-SD and RDW-CV

### DFS analysis

In the univariate analysis, RDW-SD ≤ 40.2 fl (p = 0.047), T3-T4 stage (p = 0.017), lymph node metastasis (p < 0.001), multiple tumors (p = 0.018), preoperative CEA > 5 ng/mL (p < 0.001), ALT > 50 U/L (p = 0.019) and blood loss ≥ 300 mL (p = 0.008) were associated with worse DFS. RDW-CV (p = 0.579) and SCC score (p = 0.247) were not associated with DFS (Table 4). The multivariate analysis showed that RDW-SD level was not an independent prognostic factor, and positive lymph node (HR = 2.566, 95% CI: 1.388–4.746, p = 0.003), multiple tumors (HR = 2.181, 95% CI: 1.296–3.671, p = 0.003) and preoperative CEA > 5 ng/mL (HR = 1.750, 95% CI: 1.045–2.929, p = 0.033) were independent predictors for the unfavourable OS.

**Table 4:** Prognostic factors for DFS for ICC patients in Univariate analysis

Factor	Univariate analysis		Factor	Univariate analysis	
	HR (95%CI)	Value p		HR (95%CI)	Value p
Age ≥ 60 years	0.797 (0.541–1.174)	0.251	Preoperative CEA > 5 ng/mL	2.210 (1.442–3.386)	< 0.001
Male	1.119 (0.764–1.640)	0.563	RDW-SD > 40.2 fl	0.681 (0.459–1.011)	0.047
BMI ≥ 24 kg/m^2^	1.014 (0.689–1.492)	0.945	RDW-CV > 12.6%	0.896 (0.608–1.320)	0.579
ASA score 3–4	1.352 (0.703–2.599)	0.366	ALB ≥ 40 g/L	0.744 (0.478–1.159)	0.191
Cirrhosis	1.101 (0.743–1.632)	0.630	TBIL > 21 umol/L	0.912 (0.488–1.703)	0.772
HBsAg ( +)	1.193 (0.765–1.858)	0.436	AST > 40 U/L	1.606 (0.898–2.873)	0.111
T3-T4 stage	1.711 (1.100–2.662)	0.017	ALT > 50U/L	2.018 (1.120–3.634)	0.019
Lymphnode metastasis		< 0.001	Operation time ≥ 230 min^a^	1.380 (0.941–2.022)	0.099
Undissected lymph node	Reference		Blood loss ≥ 300 ml^a^	1.710 (1.149–2.546)	0.008
Negative lymph node	1.114 (0.685–1.811)	0.664	Major liver resection	1.402 (0.906–2.171)	0.129
Positive lymph node	2.713 (1.591–4.627)	< 0.001	Post-operative Complications	0.998 (0.674–1.477)	0.991
Tumor size ≥ 5 cm	1.285 (0.877–1.884)	0.199	Preoperative therapy	1.207 (0.587–2.484)	0.609
Multiple tumors	1.738 (1.099–2.751)	0.018	Adjuvant therapy	0.887 (0.602–1.306)	0.544
Tumor location (central tumor)	0.702 (0.480–1.028)	0.069	*The prognostic value on SCC score*		0.247
Tumor location (right lobe)	0.906 (0.672–1.222)	0.518	SCC = 0	Reference	
Poor differentiation	1.443 (0.965–2.158)	0.074	SCC = 1	0.716 (0.447–1.146)	0.164
Preoperative CA19-9 > 27 U/mL	1.364 (0.875–2.124)	0.170	SCC = 2	0.698 (0.438–1.113)	0.131

^a^The median operation time and the median blood loss were chosen as the cut-off point

## Discussion

To the best of knowledge, this study is the first to use X-tile software to objectively identify the optimal cutoff point values of RDW-SD and RDW-CV for predicting survival. We discussed the relationship between preoperative RDW values and postoperative prognosis in ICC patients on the premise of excluding other diseases that affect RDW value. Our results indicated that patients with higher RDW values had better prognoses.

RDW level is an marker of the degree of erythrocyte morphology imbalance in the blood, and it reflects the heterogeneity of erythrocyte volume [26]. RDW level may change as a result of blood diseases, infectious diseases, and even cancer [27, 28]. Additionally, studies have revealed that RDW level was an independent risk factor for survival in patients with colorectal cancer and bladder cancer [29–31]. RDW values are reflected in coefficient of variation of red cell volume distribution width (RDW-CV) and standard deviation of red cell volume distribution width ((RDW-SD) levels. RDW-SD and RDW-CV were generally used to evaluate the RDW level in clinics. In our study, patients with high RDW-SD or RDW-CV were significantly associated with better OS. We used SCC as the combined index of RDW-SD and RDW-CV, and compared with SCC = 0 or SCC = 1, SCC = 2 was significantly associated with better OS and had an equivalent DFS. SCC is a combined indicator, so the differences between the three classifications further strengthen their significance for predicting survival. In general, although RDW can predict the prognosis of ICC patients to some extent, its predictions seem to contradict the conclusions of mainstream studies that have shown that RDW value is an independent risk factor for overall survival in patients with HCC, endometrial cancer and prostate cancer in a multivariate analysis [18, 32, 33]. However, there are also studies that show that RDW was not an independent predictor of cancer-specific survival (CSS) or OS in other cancers [24, 34]. These controversial conclusions led us to re-examine the value of RDW in predicting tumor prognosis and reflecting the heterogeneity among different cancer species.

According to current research evidence, the mechanisms for the poor survival of cancers are not fully understood, are very likely multifactorial, and include inflammation, oxidative stress, and malnutrition [35–37]. However, the biological meaning of RDW increase remains largely unknown in spite of several explanations that might indicate the elevated RDW levels. RDW is positively correlated with widely used plasma inflammatory markers, such as C-reactive protein (CRP) [38, 39] and blood sedimentation rate (ESR) [40], and is considered to be an inflammatory marker in cancer patients. Various inflammatory factors affect erythropoiesis through the production of erythropoietin (EPO), the inhibition of erythro-progenitor cells, and the reduction of iron release. In conclusion, the hypothesis that RDW can reflect the inflammatory state of cancer is reasonable. Second, malnutrition is another characteristic of cancer due to loss of appetite and weight loss and can lead to deficiencies in minerals and vitamins such as iron, folic acid and vitamin B12, which can also lead to changes in RDW values. In summary, high RDW levels are a good indicator of chronic inflammation and malnutrition in cancer patients. However, it is still controversial whether the prognosis of tumor patients can be predicted in a real-world setting. Studies point to evidence that tumors mainly occur in middle-aged and elderly populations, and the prevalence of anemia and malnutrition in elderly patients is relatively high, which may lead to elevated RDW values due to other aspects, thereby reducing the prognostic significance of this parameter for tumors [20]. Similarly, the investigators demonstrated that RDW was no longer associated with OS or DFS in patients with esophageal squamous cells after adjusting the correlation index [41]. Another study of esophageal cancer reached the same conclusion [42]. Such differences in outcome may be related to the selected population. The inclusion criteria for patients in this study included no association with chronic pneumonia or other diseases that affect RDW value, thus excluding the influence of these diseases on RDW and the analysis results. Most of the patients in this study had RDW values within the normal range, while other studies, especially those investigating blood-related diseases, did not exclude patients with a large number of RDW abnormalities, so different results may appear. In addition, there was heterogeneity among different cancer species, and the predictive value of RDW may not be applicable to all disease species. Among the differences in statistical methods, the heterogeneity of RDW in different studies led to different methods for selecting cut-off values. The majority of the studies used ROC analysis to define the cut-off values. In some studies, the median was used as the cut-off point, and the above two methods were used to select a cut-off point value of 90% with an applied cut-off value between 13 and 15% [43]. In this study, X-tile analysis was used for the first time to obtain a cut-off point value of 12.6% by fitting the relationship between prognosis and RDW. The differences in statistical methods led to different research results.

There are also limitations in this study. First, only preoperative RDW values were included in this study, but the clinical value of postoperative RDW remains unclear and may dynamically represent changes in the balance between systemic inflammatory responses and immune responses after treatment. Second, this study revealed that pre-operative CA19-9 level was not significantly associated with survival, the reason of which was that some patients with concomitant cholangitis (complicated by symptoms such as epigastric pain, hyperleukocytosis, or fever) were included in this study. Some studies have shown that preoperative cholangitis may affect on the value of CA19-9 [44, 45]. In future studies, in order to remove the influence of cholangitis on CA19-9 or other markers, we would strictly include the criteria and exclude this subset of patients. Finally, the data were collected retrospectively from a single center; therefore, our results may be potentially biased and inaccurate.

## Conclusions

In conclusion, our results indicated that elevated preoperative RDW values within a certain range do not indicate a worse prognosis; more meaningful results were obtained: we obtained the opposite conclusion as that in the literature. However, the significance of RDW in predicting the prognosis of ICC needs to be confirmed by larger, prospective, randomized studies.

## Data Availability

The data that support the findings of this study are available from the corresponding author upon reasonable request. Emails could be sent to the address below to obtain the shared data: lxc_pumc@126.com.

## Abbreviations

ICC: Intrahepatic cholangiocarcinoma
OS: Overall survival
PLT: Platelet count
NLR: Neutrophil–lymphocyte ratio
PLR: Platelet–lymphocyte ratio
RDW: Red blood cell distribution width
RDW-SD: Red blood cell distribution width-standard deviation
RDW-CV: Red blood cell distribution width-coefficient of variation
SCC: RDW-SD combined with RDW-CV
HCC: Hepatocellular carcinoma
CA19-9: Carbohydrate antigen 19-9
CEA: Carcinoembryonic antigen
AFP: Alpha fetoprotein
MRI: Magnetic resonance imaging
CT: Computed tomography
MVI: Microvascular invasion
ALB: Albumin
TBIL: Total bilirubin
AST: Aspartate aminotransferase
ALT: Alanine aminotransferase
TACE: Transarterial chemoembolization
HBsAg: Hepatitis B surface antigen
DFS: Disease-free survival
CRP: C-reactive protein
ESR: Blood sedimentation rate
EPO: Erythropoietin
